# Systematic review of chronic ankle instability in children

**DOI:** 10.1186/1757-1146-7-21

**Published:** 2014-03-19

**Authors:** Melissa Mandarakas, Fereshteh Pourkazemi, Amy Sman, Joshua Burns, Claire E Hiller

**Affiliations:** 1 Arthritis and Musculoskeletal Research Group, Faculty of Health Sciences, The University of Sydney, PO Box 170, Lidcombe, NSW 1825, Australia; 2 Institute for Neuroscience and Muscle Research, The Children’s Hospital at Westmead, Sydney NSW, Australia

**Keywords:** *Ankle*, *pediatrics*, *joint instability*, *sprains and strains*

## Abstract

**Background:**

Chronic ankle instability (CAI) is a disabling condition often encountered after ankle injury. Three main components of CAI exist; perceived instability; mechanical instability (increased ankle ligament laxity); and recurrent sprain. Literature evaluating CAI has been heavily focused on adults, with little attention to CAI in children. Hence, the objective of this study was to systematically review the prevalence of CAI in children.

**Methods:**

Studies were retrieved from major databases from earliest records to March 2013. References from identified articles were also examined. Studies involving participants with CAI, classified by authors as children, were considered for inclusion. Papers investigating traumatic instability or instability arising from fractures were excluded. Two independent examiners undertook all stages of screening, data extraction and methodological quality assessments. Screening discrepancies were resolved by reaching consensus.

**Results:**

Following the removal of duplicates, 14,263 papers were screened for eligibility against inclusion and exclusion criteria. Nine full papers were included in the review. Symptoms of CAI evaluated included perceived and mechanical ankle instability along with recurrent ankle sprain. In children with a history of ankle sprain, perceived instability was reported in 23-71% whilst mechanical instability was found in 18-47% of children. A history of recurrent ankle sprain was found in 22% of children.

**Conclusion:**

Due to the long-lasting impacts of CAI, future research into the measurement and incidence of ankle instability in children is recommended.

## Background

Chronic ankle instability (CAI) is a debilitating condition commonly encountered after ankle injury [1]. Three main components of CAI exist; perceived instability, mechanical instability and recurrent sprain [2]. People may experience one, two or all of these components. Perceived instability often involves the feeling that the ankle gives way and/or is unsteady during activity, weaker or less functional when compared to a steadier ankle, or prior to injury [1,3]. The perception that the ankle joint is unsteady is thought to be associated with impairments in neuromuscular and postural control, making the ankle vulnerable to repeated sprain [4,5].

CAI is common, with many adults enduring negative impacts long into the future. Following ankle sprain, up to 32% of people will develop CAI [6]. Of these, 72% will have their function impaired [6]. Activity is often impacted by the symptom of recurrent sprain, causing 18% of people to report a decreased ability to play sport, and 11% to be unable to walk long distances [7]. CAI leads to changes in, or the cessation of, sporting and occupational activities [6,8].

Research to date has been heavily focused on adults and there appears to be little attention on the prevalence of CAI specific to the pediatric population. The limited body of research on CAI in children reports that it is commonly suffered by children following sporting injuries [9], hypermobility [10] and in those with inherited neuropathies such as Charcot Marie Tooth disease (CMT). CMT is a peripheral nerve disease which commonly inflicts symptoms of a cavus foot deformity, muscle atrophy, decreased sensation, and peripheral weakness [11,12]. This results in ankle unsteadiness, causing trips, falls and ankle sprain injuries [13].

Due to the long lasting impacts CAI inflicts on the quality of life and activity of adults, it is important to investigate CAI in children [6-8]. With deeper understanding of CAI in children, targeted early intervention strategies may be developed to prevent prolonged suffering of symptoms [6-8]. Therefore the objective of this paper was to systematically review the prevalence of CAI in children.

## Methods

### Inclusion criteria

To be eligible for inclusion studies must have focused on CAI, commonly defined as experiencing perceived instability, mechanical instability or recurrent sprain [4,14], although papers reporting any long-term problems following ankle sprain were included. All study types were included, with the exception of single case studies and narrative and systematic reviews. There were no language restrictions. Participants aged up to 18 years old were included along with studies including participants classified by the author(s) as children.

### Exclusion criteria

Studies investigating ankle instability following fractures to bones of the ankle joint were excluded from the review. Papers including a mixed sample of children and adult participants were excluded if authors could not provide original, separated data sets.

### Search strategy

Studies were retrieved from electronic databases, from inception until March 2013 including: Medline, Web of Science, Cochrane, SCOPUS, PubMed, SPORTDiscus, CINAHL and Embase. Additionally, reference lists of included studies were examined for any additional studies that met the inclusion criteria. Table 1 illustrates the search strategy utilised for the Medline database, which was modified for each database.

**Table 1:** Medline search strategy

**#1: Terms combined with ‘OR’**	**#2: Terms combined with ‘OR’**			**#3: Terms combined with ‘OR’**
The ankle	Instability	Injury	Diagnosis and measurement	Children
Ankle	Ankle instability	Sprains and strains	Instability measurement	Child
Ankle joint	Chronic instability	Inversion sprain	Measurement	Paediatric
Talocrural		Inversion injury	Instability diagnosis	Pediatric
Talocalcaneal	Chronic	Repeated sprain	Diagnosis	Boy
Tibiotalar	Joint instability	Repeated injury	Laxity	Girl
Talofibular	Mechanical instability	Recurrent sprain		Adolescent
High ankle	Functional instability	Recurrent injury		Teen
	Perceived instability	Wounds and injury		Teenager
	Unstable	Syndesmosis		Youth
		Lateral ligament, ankle		Young
		Collateral ligament		
		Talofibular ligament		
		Calcaneofibular ligament		
**Combined search: [#1 AND #2] AND #3**				

Three authors [15-17] were contacted to provide original data sets for analysis. Authors were contacted to obtain additional data for a variety of reasons including; combined scores for foot and ankle problems were reported [15], data for children and adults were reported together [16] or additional baseline data for ankle instability was not provided [17]. One author responded, providing original datasets for the study by Hiller *et al.*[17] regarding the laxity and sprain history of participants.

### Assessment for study inclusion

Two independent examiners (MM and either AS or FP) screened titles, abstracts and full texts of papers according to eligibility criteria. Discrepancies were settled by a consensus, or if necessary, an additional examiner (CH).

### Methodological quality assessment

Methodological quality was assessed using a modified Downs and Black’s [18] checklist for randomized and non-randomized studies of health care interventions. Criteria assessed included: clear descriptions of the aims, outcome measures and participants, correct reporting and statistical analysis of results, the representativeness and groupings of participants, and reporting of dropouts [18]. The assessment tool provided a score out of 14. Some criteria such as participant blinding were irrelevant to some studies, hence scores were represented as a percentage for comparisons to be made. Two independent examiners assessed and rated studies (MM and either AS or FP). After independent review, discrepancies were settled by consensus. When consensus could not be reached, an additional examiner evaluated the quality to reach a final decision.

## Results

**Figure 1:**
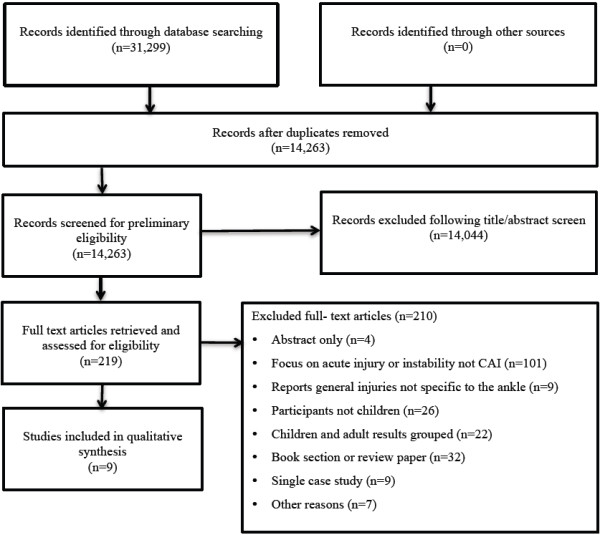
Study selection flow diagram.

Initial searching resulted in 31,299 papers. Following the removal of duplicates, the titles and abstracts of 14,263 papers were screened for potential eligibility. After initial screening, 219 articles were identified as potentially eligible and full texts were sought. Succeeding full text review and the translation of a German paper [19], nine full papers were included in the review (Figure 1) [17,19-26]. Papers were grouped according to the components of CAI investigated including: 1. Perceived instability, reporting data on patient’s perceptions of the ankle joint as being less functional, weak or painful; 2. Mechanical instability, involving measures of ligamentous laxity of the ankle; and 3. Recurrent sprain. Symptoms of CAI including perceived and mechanical ankle instability were explored in five studies [17,19,20,22,24] and prevalence of recurrent sprain in four studies [17,21,23-26] (Tables 2 and 3).

**Table 2:** Included studies in qualitative synthesis

**Author, year**	**Study type**	**Participants**	**Follow up**	**Sample size**	**Measurement of CAI**	**Epidemiology of CAI- prevalence/distribution**
**Hiller *et al. 2008*** [17]	Prospective cohort	Adolescent dancers 14.2 ± 1.8 yrs	13 months	116	Ankle instability (CAIT)	36% of all dancers unstable
						71% of sprainers unstable
					Ankle joint laxity (mod ant draw)	37% right, 47% left ankles moderate to very lax
					Self report	50% of total had history of sprain
						22% of total had history of ≥2 sprains
						38 sprains were sustained by 33 participants
						Incidence of sprains 0.21/1000 hours of dancing
**Hollwarth *et al. 1985*** [19]	Retrospective	Patients with high ankle sprain, severe trauma for inclusion	6 yrs	96	Subjective complaints; rolling over, pain, swelling, meterosensitivity	31.3% subjective complaints
		16 (range: 9–21) yrs			X-ray (AP and lateral) injured side, talar tilt stress x-ray both sides	17.7% ligament avulsions
					Ligament stiffness, pain during supination or palpation of, fibular ligaments or syndesmosis	38.5% “pathologic clinical findings”
					Abnormal talar tilt (> 5 deg)	42% abnormal
**Marchi *et al. 1999*** [20]	Prospective cohort	Patients with moderate to severe ankle injury 6–15 yrs. 26 female (48%)	3 yrs	220	Medical report of objective (limited joint mobility, pain on pressure, axial deviations, weakness, or shortening of a limb) and subjective (pain at rest or during exercise, sense of unsteadiness, or paraesthesia) symptoms	42% had objective or subjective symptoms (3 yrs follow up)
			12 yrs	54		23% had permanent symptoms (Risk ratio: 1.79, p = 0.10) (12 yrs follow up)
**Soderman *et al. 2001*** [21]	Prospective cohort	Adolescent female soccer players 15.9 ± 2.1 (range: 14–19) yrs	1 season	153	Medical report of re-injuries	56% of sprainers had recurrent sprain
**Steffen *et al. 2008*** [22]	Prospective cohort	Female soccer players 15.4 ± 0.8 (range: 14–16) yrs	-	1430	Self report of sprain history	Players with previous ankle injury (PI) more likely to sustain new ankle injury than those without (NH) (Rate ratio = 1.2 [1.1; 1.3] p < .001).
					FAOS	92.0 ± 11.3 (PI), 97.3 ± 6.0 (NH) mean difference: −5.3 (95% CI = −6.0 to −4.5)
					Pain	62.8 ± 11.1 (PI), 68.2 ± 9.7 (NH) mean difference: −5.4 (95% CI = −6.3 to −4.5)
					Symptoms	96.3 ± 7.5 (PI), 98.7 ± 4.2 (NH) mean difference: −2.3 (95% CI = −2.9 to −1.8)
					Activities of daily living	89.0 ± 16.2 (PI), 96.3 ± 8.4 (NH) mean difference: −7.3 (95%CI = −8.4 to −6.2)
					Sport and recreation function	71.3 ± 12.4 (PI), 76.3 ± 10.0 (NH) mean difference: −5.0 (95% CI = −5.9 to −4.0)
					Ankle-related quality of life	411.5 ± 46.8 (PI), and 436.7 ± 26.8 (NH) mean difference: −25.2
						(95% CI = −28.5 to −21.9)
**Swenson *et al. 2009*** [23]	Descriptive epidemiology study	High school students	-	100 high schools 13755 injuries	Medical report of re-injury	Ankle most frequently diagnosed site for recurrent injury in basketball (boys: 58.4%, girls: 43.6%), volleyball (42.7%), soccer (boys: 34.8%, girls: 37.2%), football (29.8%), softball (26.3%), and wrestling (20.1%)
						28% of all recurrent injuries were ankle injuries
						More recurrent (28%) than new ankle injuries (19%) (Injury Proportion Ratio = 1.47; 95% CI, 1.31-1.65)
**Timm *et al. 2005*** [24]	Prospective cohort	Emergency department patients with ankle injury	6 weeks	199	Medical report of:	
					Pain with activity	24 (34%) OW, 14 (15%) NW, RR = 2.25 (95% CI = 1.25-4.02)
		Range: 8–18 yrs			Persistent swelling and/or weakness	22 (31%) OW, 12 (13%) NW, RR = 2.40 (95% CI = 1.28-4.52)
					Re-injury	17 (24%) OW, 14 (15%) NW, RR = 1.60 (95% CI = 0.84-3.01)
		OW mean age = 13.9 yrs	6 months	171	Pain with activity	19 (41%) OW, 19 (16%) NW, RR = 2.57 (95% CI = 1.50-4.39)
		NW mean age = 13.5 years.			Persistent swelling and/or weakness	16 (34%) OW, 18 (15%) NW, RR = 2.28 (95% CI = 1.28-4.08)
					Re-injury	12 (26%) OW, 19 (16%) NW, RR = 1.62 (95% CI = 0.86-3.06)
						31 (44%) of OW had persistent ankle symptoms at 6 months compared with 24 (26%) NW (RR, 1.70; 95% CI, 1.10-2.61)
**Tyler *et al. 2006*** [25]	Cohort study	Male high school football players	3 seasons	152	Medical report of sprain history	50 (33%) had history of previous ankle sprain 15 non-contact ankle sprains were incurred. Of the 11 players who had a previous ankle sprain and sustained a noncontact sprain in this study, 9 (82%) injured the same ankle (incidence 2.1)
**Weir & Watson 1996**[26]	Prospective cohort	Physical education students	1 yr	266	Self report of injuries	230 injuries were incurred. The most common injuries were ankle sprains.
		Males (56%): 14.3 ± 0.85 (range: 12–15) yrs				7 overuse injuries of the ankle were incurred. 100% of overuse injuries of the ankle were re-injuries.
		Females: 14.1 ± 0.90 (range: 12–15) yrs				

KEY: CAI = Chronic Ankle Instability, CAIT = Cumberland Ankle Instability Tool, FAOS = Foot and Ankle Outcome Score, Mod ant drawer = modified anterior drawer test, OW = Children who are Overweight (≥85^th^ BMI percentile), NW = children who are of Normal Weight (<BMI 85^th^ percentile).

**Table 3:** Overview of included studies regarding components of chronic ankle instability investigated

**Component of CAI investigated**	**Author**	**Participant number**	**Participant characteristics**	**Measurement**	**Outcome**
Perceived instability	Hiller *et al.*[17]	116	Adolescent dancers	CAIT	71% of sprainers unstable
	Hollwarth *et al.*[19]	96	Severe ankle trauma	Self report	31% had complaints
	Marchi *et al.*[20]	220	Moderate-severe ankle injury	Medical report	42% had complaints 3 yrs post injury
		54			23% had complaints 12 yrs post injury
	Steffen *et al.*[22]	1430	Adolescent soccer players	FAOS	Lower function in previously injured than with no previous injury at baseline (mean diff = −25 (95% CI = −28.5 to -21.9)
	Timm *et al.*[24]	99	Patients with ankle injury	Medical report	34% had complaints
					44% of overweight children (BMI > 85^th^ percentile)
Mechanical instability	Hiller *et al.*[17]	116	Adolescent dancers	Mod ant drawer	37% Right, 47% Left of all ankles moderate to very lax
	Hollwarth *et al.*[19]	96	Severe ankle trauma	X-ray	18% had ligament avulsion
				Clinical tests	39% had pathologic clinical findings (as defined by authors)
				Talar tilt >5°	42% of total had abnormal talar tilt
Recurrent sprain	Hiller *et al.*[17]	116	Adolescent dancers	Self report	22% had ≥2 sprains
	Soderman *et al.*[21]	153	Adolescent soccer players	Medical report	56% of sprainers had recurrent sprain
	Swenson *et al.*[23]	13755 injuries	High school students	Medical report	25% of all recurrent injuries were ankle injuries
	Timm *et al.*[24]	199	Patients with ankle injury	Self report	26% of overweight (BMI > 85^th^ percentile) and16% normal weight reinjured
	Tyler *et al.*[25]	152	High school footballers	Medical report	15 non-contact ankle sprains incurred and 9 (60%) were re-sprains of the same ankle
	Weir & Watson [26]	266	Physical education students	Self report	100% overuse ankle injuries were re-injuries

KEY: BMI = Body Mass Index, CAI = Chronic Ankle Instability, CAIT = Cumberland Ankle Instability Tool, CI = Confidence Interval, FAOS = Foot and Ankle Outcome Score, Mean Diff = Mean Difference.

### Quality

The average quality of the papers was high, meeting 80.4% of the criteria (range: 38-100%, Table 4). Six papers [20-23,25,26] did not blind assessors as this was inappropriate to the study design. Other criterion commonly unfulfilled was the reporting of exact *p* values [19,21,23,25,26] and the number of participants lost to follow up [22,25,26].

**Table 4:** Results of modified Downs and Black’s quality assessment tool

**Study**		**Criteria**															
**Author**	**Year**	**1 Hypotheses/objectives**	**2 Outcomes**	**3 Participants**	**4 Findings**	**5 Data distribution**	**6 p value**	**7 Participant Selection**	**8 Represent-activeness**	**9 Blinding**	**10 Statistics**	**11 Outcome measures**	**12 Intervention groups**	**13 Time period**	**14 Follow up**	**Total**	**Percentage (%)**
Hiller *et al.*	2008	1	1	1	1	1	1	1	1	1	1	1	N/A	N/A	1	12/12	100
Hollwarth *et al.*	1985	1	0	0	1	1	0	0*	0*	0*	0*	0*	1	1	N/A	5/13	38
Marchi *et al.*	1999	1	1	1	1	1	1	1	0	0	1	1	N/A	N/A	1	10/12	83
Soderman *et al.*	2001	1	1	1	1	1	0	1	1	0	1	1	N/A	N/A	1	11/12	92
Steffen *et al.*	2008	1	1	1	1	1	1	1	1	0	1	1	N/A	N/A	0*	10/12	83
Swenson *et al.*	2009	1	1	1	1	1	0	1	1	0	1	1	N/A	N/A	N/A	9/11	82
Timm *et al.*	2005	1	1	1	1	1	1	1	1	1	1	1	1	1	1	14/14	100
Tyler *et al.*	2006	1	1	0	1	1	0	1	1	0	1	1	1	1	0*	10/14	71
Weir & Watson	1996	1	1	1	1	1	0	1	1	0	1	1	N/A	N/A	0*	9/12	75

KEY:

1 Criteria met.

0 Criteria not met.

*Unable to determine, scored 0.

N/A Criteria did not apply to study type.

### Perceived instability

Five papers investigated perceived instability including pain and impaired ankle function [17,19,20,22,24]. Symptoms of ankle instability were investigated in specific populations including dancers [17], soccer players [22], children who were overweight [24], or who had experienced “severe ankle trauma” (undefined by authors) [19,20]. Symptoms of perceived instability, pain, weakness, swelling or paraesthesia were investigated in dancers [17] and in children following ankle injuries [20,24]. Ankle injuries were self-reported via recall or diagnosed by a medical practitioner. Perceived instability was measured using tools including the Cumberland Ankle Instability Tool (CAIT) [14] and the Foot and Ankle Outcome Score (FAOS) [22], and through medical [20,24] and self reports [19].

Perceived instability and impaired ankle function during activity was common. Prevalence of perceived instability ranged from 31% in children with severe ankle injuries [19] to 71% of children who were dancers [14] (Table 3). The risk of perceived ankle instability was greatest for children who were overweight (≥ 85^th^ percentile for Body Mass Index [BMI]) [24] of a younger age [20] and in those with abnormal talar tilt [19]. For every unit increase of BMI, the risk of having long term symptoms of instability increased 0.66% (OR, 1.07; 95% CI, 1.02-1.12; p = 0.01) [24]. Of note, “permanent symptoms” of instability (lasting up to 12 years) were more frequent for injuries sustained by children under 10 years, compared to children aged over 10 years who were prone to more temporary symptoms (lasting 3 years, p < 0.05) [20]. Subjective complaints of poor ankle functioning were most notable in those who had a history of ankle injury [23] and in children following severe ankle trauma with abnormal talar tilt (>5°) [19].

### Mechanical instability

Mechanical instability following ankle sprain was investigated in two studies using four measures. Prevalence of mechanical instability was between 18% of children following severe ankle trauma [19] and 47% children who were dancers [17] (Table 2). A modified anterior drawer test identified increased laxity in adolescent dancers to be associated with lower CAIT scores, indicative of higher ankle instability (r = −0.484, p < 0.01) [17]. Stress x-ray and talar tilt revealed a high prevalence of abnormal talar tilting (>5°) in 42% of children six years after severe ankle trauma [19].

### Recurrent sprain

Six studies investigated recurrent sprain or re-injury rates using self or medical reports (Table 3) [17,21,23-26] using self [17,24,26] or medical reports [21,23,25]. A history of sprain ranged from 22% in football players [25] to 50% in dancers [17]. Only one study reported results of recurrent ankle sprain across a population, finding 22% of dancers had a history of recurrent sprain [17] (Table 2). The prevalence of recurrent sprain in children who had previously sprained their ankle ranged from 16% of normal weight children presenting to an emergency department for an ankle injury and 100% of ankle injuries sustained by physical education students [26]. Overweight children experienced a higher incidence of re-injuries to the ankle than those of normal weight (Table 2) [24].

## Discussion

CAI is a problem in the pediatric population as illustrated by the high prevalence of perceived instability, mechanical instability and recurrent sprain in specific groups of children with: past ankle injuries [19,20,23,26], dancers [17], soccer players [21,22], and those with a high BMI [24,25].

The prevalence of perceived instability was as high as 71% in children following ankle injury across the specific populations studied, with many participants reporting symptoms lasting up to 12 years [20]. Characteristics of perceived ankle instability via self-reporting of symptoms were noted in dancers [17], soccer players [22] and children who were overweight [24]. A systematic review of ankle sprains in adults reports a prevalence of perceived instability following acute ankle sprain ranging between 7% and 53% [27]. Perceived ankle instability in particular has been shown to have a large impact in adults, leading to changes in sporting and occupational activities [8]. The prevalence found in children is higher than the reported prevalence in adults. This may not be reflective of a true difference due to different testing methodologies employed. Perceived instability was measured with adult questionnaires including the CAIT and the FAOS, or the recording of subjective complaints. No pediatric-specific tool was available to measure this construct in children, which may account for the higher rate as items may have been misunderstood. Improving the measurement of perceived ankle instability in children would allow for any discrepancies due to questionnaire misinterpretation to be eliminated.

Alternatively, the experiences of adults compared to children with CAI may differ depending on the age of the first ankle sprain encountered and the onset of CAI. The age of the first ankle sprain endured by adults is rarely reported in the literature. Hence, it is unknown if adults with long-term symptoms of perceived instability incurred their first sprain as a child or as an adult. The lower prevalence of perceived instability observed in adults might be unique to this older age group if their first ankle sprain leading to CAI was recent, during adulthood.

The prevalence of mechanical instability was reported to be as high as 47% in children who were dancers using the anterior drawer test [17] and in 42% of children using stress x-ray [19]. In previous reports, 25% of adults who had experienced lateral ankle sprain within six months prior to the study were found to be ‘moderately lax’ with an anterior drawer test [28]. The higher occurrence of mechanical instability in dancers may be due to the increased general joint laxity in this population [29].

Prevalence of recurrent sprain was high across most groups of children and adolescents studied. Medical and self-reports highlighted that in up to 100% of participants who experienced an ankle injury, it was a re-injury to the joint [26]. This is higher than reports in adult research, where the incidence of re-sprain following an acute ankle sprain was as high as 34% [27]. Increased prevalence in children may be reflective of the specific active populations studied in the review, such as dancers and soccer players. Ankle sprain is commonly experienced, accounting for up to 37% of injuries in children’s soccer [23]. Therefore, this high prevalence in children of specific sporting groups may not reflect the true prevalence across all children and activity levels, perhaps making the prevalence rate more comparable to that of adults.

It was observed in the literature that like adults, children with a previous ankle injury such as ankle sprain are more likely to injure their ankles compared to those with no history of injury [17,21-23]. Up to 52% of adults with recurrent sprain have problems lasting longer than 10 years [7]. As a result of recurrent injury, up to 73% of adults with recurrent sprain injuries suffer from symptoms of pain, with 77% experiencing weakness [7]. The high prevalence of recurrent ankle sprain in children is therefore of particular concern. Following moderate to severe ankle injury, such as ankle sprain, children under the age of 10 years have been shown to be more likely to develop long-term symptoms than children over 10 years of age [20]. With such symptoms induced so early in life, and the impact of these symptoms on activity levels and quality of life, recurrent sprain poses a threat to the health and wellbeing of children as they grow and develop.

The review demonstrated that children commonly show signs and symptoms of the multiple components of CAI. An analysis of additional, unpublished data collected as part of a study by Hiller *et al.*[17] showed that an increased number of ankle sprains and increased laxity were found to be significantly associated with decreased CAIT scores (indicating greater instability). This finding highlights a relationship between perceived and mechanical aspects of ankle instability in addition to recurrent sprain [30]. However, while mechanical and perceived ankle instability, along with recurrent sprain may be linked, it is not necessary to experience symptoms across all of these domains. Hollwarth *et al*. [19] found that the absence of mechanical instability did not necessarily prevent the experience of perceived instability. These findings may be attributed to the initial trauma to the ankle in participants causing deficits in balance, strength, proprioception and joint position sense; leading to the experience of perceived instability without showing signs of mechanical instability [2].

To measure components of CAI, adult tools were often utilised including the CAIT and FAOS. No tool was found that had been developed or validated specifically for pediatric use to measure domains of CAI. Whilst adult tools may be appropriate to measure mechanical instability and recurrent sprain, they might be inappropriate to measure perceived instability. Due to the reflective nature of the construct, measurement is by self-report of complaints. Hence, questionnaires must be carefully developed and tested for their readability and comprehension by children to gain true insight into the perception of their ankle functioning. The Cumberland Ankle Instability Tool was recently modified to create the CAIT-Youth (CAITY) for use with a pediatric population [31]. The CAITY was developed and validated in children aged eight to sixteen years to measure perceived ankle instability. We recommend the use of this tool in future studies as a measurement tool for ankle instability in children [31].

A limitation of this review was that many papers that did investigate CAI were excluded due to the grouping of the results of children and adults together. Therefore, more information on CAI in children may be available than could be extracted for review due to the inclusion criteria of utilised for the present study.

Future research with a focus on CAI in the general pediatric community is recommended, in addition to specific sporting, clinical or post-injury populations. This may reveal the true incidence of CAI in children irrespective of physical activity, BMI and history of severe injury.

## Conclusion

The prevalence of CAI was high in specific groups of children and adolescents studied, comparable and often higher to that of adult populations. However, this systematic review found a limited volume of research about CAI in children. As no tool of high quality existed to measure perceptual components of CAI in children, the prevalence, distribution and impact of CAI on children was difficult to determine. Future research into CAI in children is recommended to bridge this gap between clinical knowledge and evidence in the literature.

## Competing interests

The authors declare that they have no competing interests.

## Authors’ contributions

MM participated in the conception and design of the study, the collection, selection and review of papers and drafted the final manuscript. FP and AS were involved in the screening, selection and review of papers. JB was involved in the conception and design of the study and critically revised the manuscript for important intellectual content. CH was involved in the conception and design of the study, reviewing the papers and critically revised the manuscript for important intellectual content. All authors read and approved the final manuscript.
